# The Effect of a Higher Bias Gas Flow on Imposed T-Piece Resistance and Breathing in Preterm Infants at Birth

**DOI:** 10.3389/fped.2022.817010

**Published:** 2022-03-17

**Authors:** Kristel L. A. M. Kuypers, Lieve A. Willemsen, Sophie J. E. Cramer, Aidan J. Kashyap, Thomas Drevhammar, Stuart B. Hooper, Arjan B. te Pas

**Affiliations:** ^1^Division of Neonatology, Department of Paediatrics, Willem-Alexander Children’s Hospital, Leiden University Medical Centre, Leiden, Netherlands; ^2^The Ritchie Centre, Hudson Institute of Medical Research, Melbourne, VIC, Australia; ^3^Department of Obstetrics and Gynaecology, Monash University, Melbourne, VIC, Australia; ^4^Department of Women’s and Children’s Health, Karolinska Institutet, Stockholm, Sweden; ^5^Department of Anaesthesiology, Östersund Hospital, Östersund, Sweden

**Keywords:** neonatal resuscitation, preterm infants, imposed resistance, T-piece, bias gas flow, breathing, valve resistance, continuous positive airway pressure

## Abstract

**Objective:**

The resistance created by the PEEP-valve of a T-piece resuscitator is bias gas flow dependent and might affect breathing in preterm infants. In this study we investigated the effect of a higher bias gas flow on the imposed inspiratory and expiratory T-piece resistance and expiratory breaking manoeuvres (EBM) in preterm infants during spontaneous breathing on CPAP at birth.

**Methods:**

In a retrospective pre-post implementation study of preterm infants <32 weeks gestation, who were stabilised with a T-piece resuscitator, a bias gas flow of 12 L/min was compared to 8 L/min. All spontaneous breaths on CPAP within the first 10 min of starting respiratory support were analysed on a breath-by-breath basis to determine the breathing pattern of each breath and to calculate the imposed inspiratory and expiratory T-piece resistance (Ri, Re), flow rates and tidal volume.

**Results:**

In total, 54 infants were included (bias gas flow 12 L/min: *n* = 27, 8 L/min: *n* = 27) with a median GA of 29^+6^ (28^+4^–30^+3^) and 28^+5^ (25^+6^–30^+3^), respectively (*p* = 0.182). Ri and Re were significantly lower in the 12 L/min compared to 8 L/min bias flow group [Ri: 29.6 (26.1–33.6) vs. 46.4 (43.0–54.1) cm H_2_O/L/s, *p* < 0.001; Re: 32.0 (30.0–35.1) vs. 48.0 (46.3–53.9) cm H_2_O/L/s, *p* < 0.001], while the incidence of EBM [77% (53–88) vs. 77% (58–90), *p* = 0.586] was similar.

**Conclusion:**

During stabilisation of preterm infants at birth with a T-piece resuscitator, the use of a higher bias gas flow reduced both the imposed inspiratory and expiratory T-piece resistance for the infant, but this did not influence the incidence of EBMs.

## Introduction

Respiratory support, usually in the form of either continuous positive airway pressure (CPAP) or intermittent positive pressure ventilation (iPPV), is often needed to stabilise preterm infants after birth. Currently, this is commonly performed using a T-piece resuscitation device such as the Neopuff™, which provides positive end expiratory pressure (PEEP) or CPAP when the expiratory outlet is open and positive inflation pressures (PIP) when the expiratory outlet is occluded (1). CPAP or PEEP are generated by providing resistance to flow in the expiratory outlet of the T-piece, also known as the PEEP valve (2). To set the desired PEEP/CPAP level for any given bias gas flow, the outlet that would normally direct flow to the infant is occluded and the resistance of the PEEP valve is regulated by altering its aperture. Higher bias gas flows (e.g., 12–15 L/min) require a larger PEEP valve aperture, because a lower resistance is required to achieve a set PEEP/CPAP level compared to low bias gas flows (e.g., 6–8 L/min). As the infant’s expiratory air must pass through the PEEP valve, reductions in the resistance of the valve will result in reduced expiratory resistance imposed on the infant. In theory, reduced imposed resistance should result in reduced breathing effort and energy cost, also known as the imposed work of breathing (iWOB) (2, 3).

Recently, a multicentre randomised controlled trial (4) compared a low iWOB respiratory support system with bi-nasal prongs to a high iWOB (5) T-piece system with a face mask as primary respiratory support system in the delivery room. This study reported fewer delivery room intubations when a low iWOB system was used compared to a high iWOB system. Rather than replacing the respiratory support system, we hypothesised that the iWOB may alternatively be reduced by using a higher bias gas flow to reduce the valve resistance of the T-piece.

Although we hypothesise that reducing the imposed T-piece resistance might be beneficial for preterm infants at birth, it is important to evaluate the possible consequences of a reduced expiratory resistance on the infant’s breathing pattern. At birth, preterm infants often regulate their own airflow during expiration by increasing laryngeal resistance through expiratory braking manoeuvres (EBM). They close their larynx during expiration to increase pulmonary pressure in order to promote alveolar recruitment and prevent alveolar collapse (2, 6). While several studies (7, 8) have shown that CPAP reduces EBM in preterm infants at birth, the effect of imposed resistance on EBM is unknown. A lower expiratory resistance could result in higher expiratory airflows and higher deflation rates, which could potentially lead to a loss of FRC due to the lung’s higher momentum as it reaches FRC.

Based on previous studies (2, 3, 5, 9), bias gas flows were increased from 8 to 12 L/min in the delivery room at Leiden University Medical Centre (LUMC) in June 2019 to reduce the valve and imposed T-piece resistance during neonatal stabilisation. This was consistent with the operating manual of the Neopuff™ device which recommends a gas flow range from 5 to 15 L/min (10). This retrospective pre-post implementation study aimed to investigate the effect of a higher gas flow on the imposed inspiratory and expiratory T-piece resistance and EBM in preterm infants during spontaneous breathing on CPAP at birth.

## Materials and Methods

A retrospective pre-post implementation study was performed at the LUMC, a tertiary-level perinatal centre in Netherlands. We selected infants who (i) were born <32 weeks of gestation, (ii) received respiratory support at birth, (iii) had complete respiratory function monitor (RFM) files, and (iv) maintained spontaneous breathing on CPAP for at least 1 min during the first 10 min of respiratory support. All infants were born between March and December 2019, with the higher bias gas flow setting being implemented in June 2019. Due to the transition in June, all infants born in June were excluded. Preterm infants with congenital abnormalities affecting the respiratory transition at birth were excluded. The database was scanned in chronological order and in each group, the first 27 eligible infants were included.

Respiratory support was provided by the Neopuff™ T-Piece resuscitator (Neopuff™ Infant Resuscitator, Fisher & Paykel Healthcare Ltd., Auckland, New Zealand) *via* face mask (Neonatal Resuscitation Mask, Fisher & Paykel Healthcare Ltd., Auckland, New Zealand). According to the local protocol, respiratory support started with 5–8 cm H_2_O CPAP, using a bias gas flow of 8 or 12 L/min. The fraction of inspired oxygen (FiO_2_) was initially set at 0.3 and could be titrated up based on the 25th percentile of the Dawson nomograms (11). When necessary, initial inflations of 3 s and/or iPPV were given using positive end-expiratory pressures (PEEP) of 5–8 cm H_2_O and peak inspiratory pressures (PIP) of 20–25 cm H_2_O at 40–60 inflations/min. To record SpO_2_ and heart rate, a Radical-7Masimo SET pulseoximeter probe (Masimo Corporation, Irvine, CA, United States) was placed around the infant’s right wrist. The Teledyne Oxygen Analyser AX300-I (Teledyne Analytical Instruments, City of Industry, CA, United States) inserted into the inspiratory limb of the Neopuff™ circuit measured fraction of inspired oxygen (FiO_2_), while the disposable Avea Varflex Flow transducer (Carefusion, Yorba Linda, CA, United States) connected between the Neopuff™ and the facemask measured flows and pressures. Signals were collected by the New Life BOX Neo-RDS (Applied Biosignals, Weener, Germany) connected to the RFM and saved by Polybench software (Applied Biosignals, Weener, Germany). Pulmochart software (Applied Biosignals, Weener, Germany) allowed a breath-by-breath analysis to calculate breathing parameters, corrected for birth weight (12).

The use of antenatal corticosteroids and general anaesthetics, mode of delivery, gestational age (GA), birth weight, gender, umbilical cord blood pH and Apgar score at 1, 5, and 10 min after birth were collected from the medical records of all included infants to describe baseline characteristics.

The amount of bias gas flow was measured using the RFM recordings. All spontaneous breaths on CPAP within the first 10 min of respiratory support were analysed on a breath-by-breath basis to calculate peak and mean inspiratory and expiratory flow rates (PIFR, PEFR, MIFR, MEFR), tidal volume (Vt), inspiratory and expiratory time (Ti, Te), minute volume, breathing rate and interbreath variability. When leak or measurement artifacts were present, breaths were excluded from the analysis.

The flow and volume waveforms of the RFM were used to define the breathing pattern with or without expiratory braking, as described by te Pas et al. (6, 13). The presence of leak, initial inflations and/or iPPV were noted. In addition, heart rate, oxygen saturation and fraction of inspired oxygen of the first 10 min from birth were noted.

**FIGURE 1:**
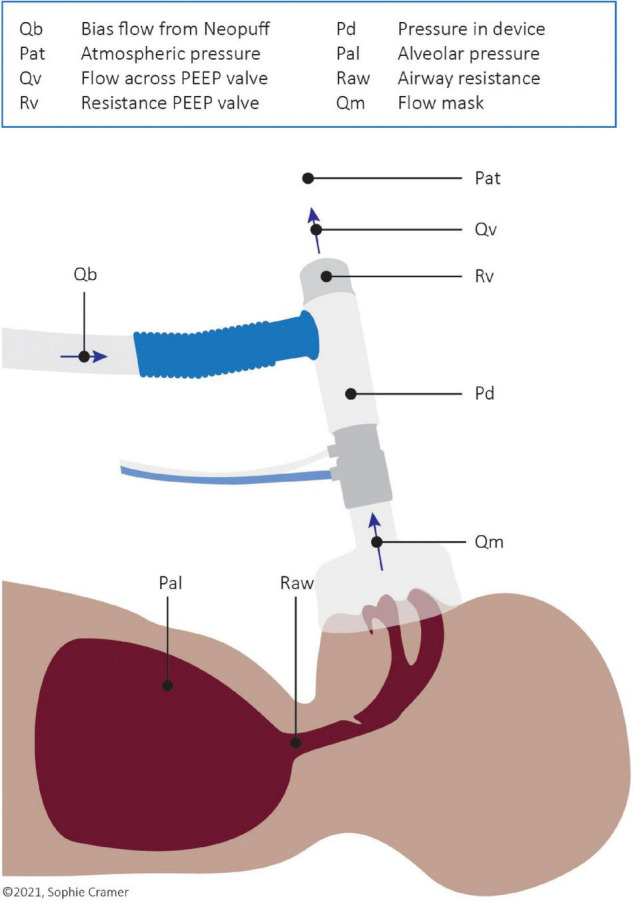
Schematic visualisation of flows, pressures, and resistance during spontaneous breathing.


The resistance of the PEEP valve (Rv) as well as the imposed inspiratory and expiratory T-piece resistance (Ri, Re) was calculated using a derivation of Ohm’s law (Eq. 1 below and Figure 1).

(1a) $$R\,v=\frac{△\,P}{Q\,v}=\frac{P\,d-P\,a\,t}{Q\,b+Q\,m}=\frac{C\,P\,A\,P-0}{Q\,b+0}$$

(1b) $$R\,i=\frac{P\,d-P\,a\,t}{Q\,b+Q\,m}=\frac{m\,i\,n\,i\,m\,u\,m\,i\,n\,p\,s\,i\,r\,a\,t\,o\,r\,y\,p\,r\,e\,s\,s\,u\,r\,e-0}{Q\,b-P\,I\,F\,R}$$

(1c) $$R\,e=\frac{P\,d-P\,a\,t}{Q\,b+Q\,m}=\frac{p\,e\,a\,k\,e\,x\,p\,i\,r\,a\,t\,o\,r\,y\,p\,r\,e\,s\,s\,u\,r\,e-0}{Q\,b+P\,E\,F\,R}$$

△*P* is the pressure difference across the PEEP valve and thus the difference between the pressure within the device (Pd) and atmospheric pressure (Pat; reference to 0 cm H_2_O). Qv is the flow through the PEEP valve, which is the sum of the bias gas flow (Qb) from the Neopuff™ and the expiratory flow through the mask (Qm). During inspiration, Qm has a negative value and so Qv equals the bias flow minus the inspiratory flow through the mask.

When CPAP is provided and no flow is entering or leaving the infant (i.e., when Qm = 0, which occurs at end-expiration or end-inspiration), 100% of the bias gas flow (Qb) will exit the T-piece *via* the PEEP valve and the pressure within the device (Pd) will equal the set CPAP/PEEP level. During expiration, all expired air leaves the system *via* the PEEP valve, resulting in an increased flow over the valve. As the resistance of the valve and the atmospheric pressure remain the same, the pressure in the device will slightly increase above the set CPAP level. The opposite effect is seen during inspiration; less flow travels through the PEEP valve and the pressure in the device will slightly decrease below the set CPAP level. These fluctuations counteract breathing as the pressure in the lung has to overcome the increased pressure in the device during expiration and the reduced pressure during inspiration. Fluctuations in CPAP were calculated for every breath using Eqs 2, 3.


(2) △CPAPinsp=PEEP-minimum⁢inspiratory⁢pressure

(3) △CPAPexp=peak⁢expiratory⁢pressure-PEEP

The local institutional Research Ethics Committee of the LUMC approved the study protocol (G19.129) and issued a statement of no objection for performing this study.

All statistical analyses were performed with IBM SPSS Statistics version 25 (IBM Software, Chicago, IL, United States, 2016). Quantitative data are presented as median (IQR), mean ± SD or *n* (%) as appropriate. Categorical data were analysed using a Chi-square test, normally distributed continuous variables using the independent *t*-test and skewed continuous variables using the Mann–Whitney test. Linear regression analysis has been performed to correct for the effect of GA and gender on the percentage of EBMs. A *p*-value < 0.05 was considered statistically significant. This is the first study to compare the expiratory resistance of a T-piece resuscitator during CPAP with different bias gas flows. Therefore, the sample size was calculated based on the expiratory resistance described by Abbasi et al. (14). This study reported a mean expiratory resistance of 94 ± 12 cm H_2_O/L/s in healthy low birthweight infants <34 weeks 0.5 week postnatally, who didn’t receive mechanical ventilation. To detect an absolute decrease of 10% from 94 cm H_2_O/L/s using a standard deviation of 12 cm H_2_O/L/s, with a power of 0.80 and an α-error of 5%, we required 54 infants in total (27 infants per group).

## Results

We recorded 107 resuscitations of preterm infants <32 weeks between March and December 2019. Fourteen infants were not eligible as they were born around the implementation date of June 2019. The first 27 infants that met the inclusion criteria were included in each group. The median (IQR) GA was 29^+6^ (28^+4^–30^+3^) for the 12 L/min bias gas flow and 28^+5^ (25^+6^–30^+3^) weeks for the 8 L/min bias gas flow group (*p* = 0.182), with significantly more males in the 12 L/min bias gas flow group (63.0 vs. 33.3%, *p* = 0.029). There were no differences in other baseline characteristics between the two groups (Table 1).

**TABLE 1:** Patients’ characteristics.

	Bias gas flow 12 L/min (*n* = 27)	Bias gas flow 8 L/min (*n* = 27)	*p*-value
Gestational age (weeks)	29^+6^ (28^+4^–30^+3^)	28^+5^ (25^+6^–30^+3^)	0.182^a^
Birth weight (g)	1370 (867–1650)	1215 (765–1554)	0.411^a^
Male gender, *n* (%)	17 (63.0)	9 (33.3)	0.029^b^
Complete course of antenatal steroids, *n* (%)	23 (85.2)	19 (70.4)	0.190^b^
General anaesthesia, *n* (%)	3 (11.1)	1 (3.7)	0.299^b^
Caesarean section, *n* (%)	13 (48.1)	9 (33.3)	0.268^b^
Umbilical arterial pH after birth	7.3 ± 0.1^†^	7.3 ± 0.1^‡^	0.357^c^
Apgar score 1 min	5 (2–8)	5 (2–7)	0.591^a^
Apgar score 5 min	8 (7–9)	8 (7–9)	0.323^a^
Apgar score 10 min	9 (9–9)	9 (8–9)	0.385^a^
Time from birth until start respiratory support (min:sec)	1:48 ± 0:42	1:42 ± 1:05	0.681^c^
Initial inflations given, *n* (%)	16 (59.3)	18 (66.7)	0.573^b^
Positive pressure ventilation given, *n* (%)	14 (51.9)	13 (48.1)	0.785^b^
Time from birth until start spontaneous breathing on CPAP (min:sec)	3:57 ± 1:50	3:36 ± 1:31	0.453^c^

*^a^Mann–Whitney U test.*

*^b^χ2 test.*

*^c^Independent samples t-test.*

*^†^12 missing values.*

*^‡^9 missing values.*

**FIGURE 2:**
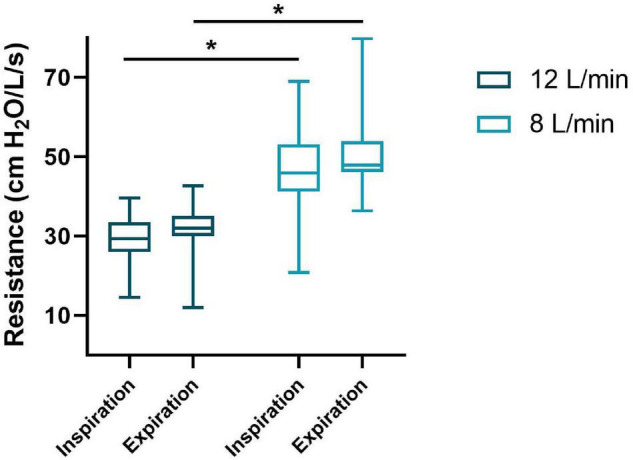
Imposed inspiratory and expiratory resistance of the T-piece during spontaneous breathing on CPAP. The median (IQR) resistance (y-axis) for inspiration and expiration of both groups (x-axis) are presented in this boxplot. *p < 0.001.

The bias gas flow (Qb) measured was 13.2 (12.8–13.9) and 9.1 (8.6–9.4) L/min resulting in Rv of 30.2 (27.9–33.5) and 47.0 (43.4–51.8) cm H_2_O/L/s in the 12 and 8 L/min flow group, respectively (Qb: *p* < 0.001, Rv: *p* < 0.001). The Ri and Re were significantly lower in the 12 L/min compared to 8 L/min bias gas flow group [Ri: 29.6 (26.1–33.6) vs. 46.4 (43.0–54.1) cm H_2_O/L/s, *p* < 0.001; Re: 32.0 (30.0–35.1) vs. 48.0 (46.3–53.9) cm H_2_O/L/s, *p* < 0.001] (Figure 2). The ΔCPAP_*insp*_ and PIFR were significantly lower in the 12 L/min bias gas flow group [ΔCPAP_*insp*_ 1.0 (0.8–1.2) vs. 1.8 (1.3–2.2) cm H_2_O, *p* < 0.001; PIFR 1.1 (0.6–1.6) vs. 1.6 (1.1–2.3) L/kg/min, *p* = 0.015], while MIFR and Vt were not different. ΔCPAP_*exp*_, PEFR, MEFR and the occurrence of EBM were not different between the groups (Table 2).

**TABLE 2:** Physiological and respiratory parameters during spontaneous breathing on CPAP.

	Bias gas flow 12 L/min	Bias gas flow 8 L/min	*p*-value^a^
Measured bias gas flow (L/min)	13.2 (12.8–13.9)	9.1 (8.6–9.6)	< 0.001
Total # breaths	281 (243–430)	283 (209–335)	0.467
# breaths without leak	233 (175–286)	198 (121–263)	0.233
**Resistance**			
Rv (cm H_2_O/L/s)	30.2 (27.9–33.5)	47.0 (43.4–51.8)	< 0.001
Ri (cm H_2_O/L/s)	29.6 (26.1–33.6)	46.4 (43.0–54.1)	< 0.001
Re (cm H_2_O/L/s)	32.0 (30.0–35.1)	48.0 (46.3–53.9)	< 0.001
**Pressure**			
CPAP level (cm H_2_O)	6.6 (6.4–7.3)	7.1 (6.7–7.7)	0.079
ΔCPAP_*ins*_ (cm H_2_O)	1.0 (0.8–1.2)	1.8 (1.3–2.2)	< 0.001
ΔCPAP_*exp*_ (cm H_2_O)	1.4 (1.0–2.0)	1.7 (1.2–2.4)	0.223
**Flow rates**			
PIFR (L/kg/min)	1.1 (0.6–1.6)	1.6 (1.1–2.3)	0.015
PEFR (L/kg/min)	1.5 (1.0–1.8)	1.5 (1.1–1.8)	0.883
MIFR (L/kg/min)	0.5 (0.4–0.7)	0.6 (0.5–0.8)	0.117
MEFR (L/kg/min)	0.5 (0.3–0.6)	0.4 (0.3–0.6)	0.528
**Respiratory time**			
Ti (sec)	0.4 (0.3–0.5)	0.4 (0.3–0.4)	0.355
Te (sec)	0.9 (0.8–1.3)	1.1 (0.8–1.3)	0.431
**Breathing pattern**			
Percentage of EBM (%)	77 (53–88)	77 (58–90)	0.586
Breathing rate (# breaths/min)	48.3 (32.0–54.2)	40.5 (33.5–53.8)	0.723
Tidal volume (mL/kg)	3.2 (2.5–4.4)	3.5 (2.7–4.9)	0.287
Minute volume (mL/kg/min)	124 (100–203)	138 (118–205)	0.431
Inter breath interval variability (%)	40.1 (34.6–47.6)	41.2 (33.7–60.6)	0.697
**Physiological parameters**			
Heart rate	144 (122–151)	135 (123.25–154.25)	0.562
SpO2 (%)	81 (75.5–83.5)	80 (76–85.75)	0.741
FiO2 (%)	45 (38.5–60)	45 (36–64)	0.898

*All values in this table are presented as median with IQR.*

*^a^Mann–Whitney U test.*

*Rv, resistance of the valve; Ri, inspiratory resistance of the valve; Re, expiratory resistance of the valve; CPAP, continuous positive airway pressure; PIFR, peak inspiratory flow rate; PEFR, peak expiratory flow rate; MIRF, mean inspiratory flow rate; MEFR, mean expiratory flow rate; Ti, inspiratory time; Te, expiratory time; EBM, expiratory breaking manouvres; SpO_2_, oxygen saturation; FiO_2_, fraction of inspired oxygen.*

To evaluate the effect of gender and GA on EBMs a regression analysis was performed with covariates bias gas flow group, gender, GA and CPAP level [*F*(4,49) = 1.429, *p* = 0.238, *R*^2^ = 0.104], which showed only a significant effect of GA on EBMs (bias gas flow group: *p* = 0.941, GA: *p* = 0.048, gender: *p* = 0.574 and CPAP level *p* = 0.254).

## Discussion

This retrospective study is the first to investigate the effect of a higher bias gas flow on imposed inspiratory and expiratory T-piece resistance and peak airflows in preterm infants during spontaneous breathing while receiving CPAP at birth. We observed that increasing bias gas flow with factor 1.5 reduced the imposed inspiratory and expiratory T-piece resistance with the same factor during stabilisation of preterm infants at birth. The reduced resistance resulted in smaller fluctuations in CPAP during both in- and expiration, indicating a decreased iWOB. Although a significantly lower PIFR was observed, MIFR, MEFR, tidal volumes, in- and expiration times, minute volumes and the incidence of EBMs remained similar.

Our study confirms the results of a bench test performed by Wald et al. (2), where increasing bias gas flow with a factor 2.5 led to the same factorial reduction in expiratory resistance for the same CPAP level. However, the effect on CPAP fluctuations (peak), inspiratory or expiratory airflows of the infant or the incidence of EBM could not be accounted for in this analysis.

The relationship between resistance and pressure fluctuations as reported in our study was also reported in a cross-over study by Hückstadt et al. (15) who compared CPAP system Infant Flow to the Babylog 8000 in preterm infants admitted to the NICU. In their study, the Infant Flow had smaller CPAP fluctuations, which is attributable to the low expiratory resistance of the device (2, 9, 15). However, they also reported an increase in PIFR and PEFR when the Infant Flow was used compared to the Babylog 8000 (15). This is in contrast with our results where the reduced resistance did not increase the peak nor the mean airflows. The different outcomes can be explained by Eq. 4 (derived from Ohm’s law, Supplementary Material and Figure 1), which shows that, assuming the airway resistance and set CPAP level remain the same, a lower resistance should lead to either higher flows (qm) or lower alveolar pressures (Pal).


(4) $$Q\,m=\frac{P\,a\,l-C\,P\,A\,P}{R\,a\,w+R\,v}$$

The results of Huckstadt (15) can be explained by assuming that both the airway resistance of the infants and the generated alveolar pressure remained the same over the study period, regardless of the device used, resulting in higher airflows when using the device with the lowest resistance.

While these assumptions might be valid in a bench test and in infants who have already successfully transitioned at birth, airway resistance and pulmonary pressures vary during the transition at birth. As the patient characteristics of both groups in our study were comparable, the inspiratory airway resistance of both groups is likely to be similar, suggesting that infants might respond differently to a reduced resistance during their transition by reducing their intrinsic effort. We were not able to measure pulmonary pressures and diaphragm activity, but we hypothesis that the infants detected a lower inspiratory resistance and reacted by expending less effort to produce an inspiration that was equally effective, as indicated by the similar Vt and minute volumes between groups. Although, the significant decrease in PIFR in the post implementation group could imply that the airway resistance was not comparable between our two groups, it is more likely that the reduced resistance led to a different flow curve given the equal mean inspiratory flows.

The fact that the expiratory airflows and the incidence of EBMs were also similar in both group, indicates that the alveolar pressure during expiration must have changed to accommodate the lower resistance. These could include: (i) the elastic recoil of the lung was counteracted by post inspiratory diaphragmatic activity or (ii) active expiration contributed to PEFR to a greater extent in infants receiving a lower bias gas flow. Another explanation could be that we recorded values at 200 Hz and so our measure of PEFR may over- or under-estimate the peak flow during expiration, as it has a steep curve at the start of expiration and a long flattened slope, particularly when expiration is active.

In summary, the different results between the study of Huckstadt (15) and ours could be explained by the differences in methodology (i.e., cross-over vs. retrospective implementation), differences in devices [resistance of Infant Flow is still over a factor 3 less than the resistance of the T-piece with 12 L/min bias gas flow (2)] and/or the timing of our study period (during transition vs. after transition at birth). We hypothesised that reducing the imposed resistance would change the breathing pattern of preterm infants as infants would (i) counteract the passive recoil using diaphragmatic post inspiratory activity and increase the pulmonary resistance, or (ii) increase their breathing rate to compensate for the lower imposed resistance to maintain FRC (6, 13). As no differences in breathing were observed in this study, it is plausible to assume that infants benefit from a higher bias gas flow in a T-piece resuscitator as this requires less effort, while still achieving effective breathing. However, as we were not able to measure the effort or airway resistance, this study does not answer the question whether or not decreasing the imposed resistance in preterm infants at birth is clinically relevant. Furthermore, the reported increased airflows by Huckstadt (15) raises the question whether further reducing or eliminating the imposed resistance would have a different impact on the infant’s breathing. Further research into the clinical relevance of (reduced) imposed resistance and/or work of breathing is needed before recommendations for neonatal resuscitation devices can be made. Nevertheless, we would suggest that a lower alveolar pressure during expiration potentially may reduce the risk of lung injury, particularly during non-synchronised iPPV.

This was a retrospective pre-post implementation study and the results should be interpreted with the appropriate caution. Pressure and flow are measured by a flow sensor in-line with the T-piece and it remains unclear if the measured pressure and flow changes are a true representation of the pulmonary pressure and flow changes. Furthermore, the resistance calculations assume laminar flow, but at higher flow rates, it is possible that some flows became turbulent.

## Conclusion

A higher bias gas flow for generating pressures with a T-piece resuscitator during stabilisation of preterm infants at birth reduced the imposed resistance created by the T-piece resuscitator. This change probably led to less effort but this did not influence EBMs of the infant. This raises the question of whether or not reducing the imposed resistance (and work of breathing) in preterm infants at birth is clinically relevant and whether eliminating the imposed resistance would have an different impact on the infant’s breathing.

## Data Availability Statement

The raw data supporting the conclusions of this article will be made available by the authors, without undue reservation.

## Ethics Statement

The studies involving human participants were reviewed and approved by Medical Ethics Committee Leiden-Den Haag-Delft. Written informed consent from the participants’ legal guardian/next of kin was not required to participate in this study in accordance with the national legislation and the institutional requirements.

## Author Contributions

KK co-conceived the study (with AP), conducted the study, collected, analysed, and interpreted the data, and wrote the first draft of the manuscript. LW contributed to data collection and analysis, and reviewed and edited the manuscript. SC, AK, and TD contributed to data interpretation, and reviewed and edited the manuscript. SH co-conceived the study, supervised the study, interpreted the data, and reviewed and edited the manuscript. AP co-conceived the study, supervised the study, interpreted the data, and reviewed and edited the first draft of the manuscript. All authors agreed to be accountable for all aspects of the work and approved the final version of the manuscript.

## Author Disclaimer

Fisher & Paykel Healthcare Limited had no role in study design nor in the collection, analysis, and interpretation of data, writing of the manuscript, and decision to submit the manuscript for publication.

## Conflict of Interest

KK is the recipient of an unrestricted research grant from Fisher & Paykel Healthcare Limited. The remaining authors declare that the research was conducted in the absence of any commercial or financial relationships that could be construed as a potential conflict of interest. The reviewer GL declared past co-authorships with the authors AP, KK and the absence of any ongoing collaboration with any of the authors to the handling editor.

## Publisher’s Note

All claims expressed in this article are solely those of the authors and do not necessarily represent those of their affiliated organizations, or those of the publisher, the editors and the reviewers. Any product that may be evaluated in this article, or claim that may be made by its manufacturer, is not guaranteed or endorsed by the publisher.

## References

[1] Hinder M McEwan A Drevhammer T Donaldson S Tracy MB. T–piece resuscitators: how do they compare? *Arch Dis Child Fetal Neonatal Ed.* (2019) 104:F122–7. 10.1136/archdischild-2018-314860 29728414

[2] Wald M Kribs A Jeitler V Lirsch D Pollak A Kirchner L. Variety of expiratory resistance between different continuous positive airway pressure devices for preterm infants. *Artif Organs.* (2011) 35:22–8. 10.1111/j.1525-1594.2010.01020.x 20618229

[3] Drevhammar T Nilsson K Zetterstrom H Jonsson B. Comparison of seven infant continuous positive airway pressure systems using simulated neonatal breathing. *Pediatr Crit Care Med.* (2012) 13:e113–9. 10.1097/PCC.0b013e31822f1b79 21946854

[4] Donaldsson S Drevhammar T Li Y Bartocci M Rettedal SI Lundberg F Comparison of respiratory support after delivery in infants born before 28 weeks’ gestational age: the CORSAD randomized clinical trial. *JAMA Pediatr.* (2021) 175:911–8. 10.1001/jamapediatrics.2021.1497 34125148 PMC8424478

[5] Donaldsson S Drevhammar T Taittonen L Klemming S Jonsson B. Initial stabilisation of preterm infants: a new resuscitation system with low imposed work of breathing for use with face mask or nasal prongs. *Arch Dis Child Fetal Neonatal Ed.* (2017) 102:F203–7. 10.1136/archdischild-2016-310577 27553588

[6] te Pas AB Davis PG Kamlin CO Dawson JA O’Donnell C Morley CJ. Spontaneous breathing patterns of very preterm infants treated with contininuous positive airway pressure at birth. *Pediatr Res.* (2008) 64:281–5. 10.1203/pdr.0b013e31817d9c35 18458652

[7] Elgellab A Riou Y Abbazine A Truffert P Matran R Lequien P Effects of nasal continuous positive airway pressure (NCPAP) on breathing pattern in spontaneously breathing premature newborn infants. *Intensive Care Med.* (2001) 27:1782–7. 10.1007/s00134-001-1117-1 11810123

[8] Drevhammer T. *Performance of nCPAP Systems for Neonatal Use and Development of a New System for Infant Resuscitation.* Stockholm: Karolinska Institutet (2016).

[9] DiBlasi RM Salyer JW Zignego JC Redding GJ Richardson CP. The impact of imposed expiratory resistance in neonatal mechanical ventilation: a laboratory evaluation. *Respir Care.* (2008) 53:1450–60. 18957147

[10] Fisher & Paykel Healthcare Ltd. *Operating Manual Neopuff™ 900 Series.* Auckland: Fisher & Paykel Healthcare Ltd (2016).

[11] Dawson JA Kamlin CO Vento M Wong C Cole TJ Donath SM Defining the reference range for oxygen saturation for infants after birth. *Pediatrics.* (2010) 125:e1340–7. 10.1542/peds.2009-1510 20439604

[12] Martherus T Kuypers KLAM Böhringer S Dekker J Witlox RSGM Hooper SB Feasibility and effect of physiological–based CPAP in preterm infants at birth. *Front Pediatr.* (2021) 9:777614. 10.3389/fped.2021.777614 34926350 PMC8678466

[13] te Pas AB Wong C Kamlin CO Dawson JA Morley CJ Davis PG. Breathing patterns in preterm and term infants immediately after birth. *Pediatr Res.* (2009) 65:352–6. 10.1203/PDR.0b013e318193f117 19391251

[14] Abbasi S Bhutani VK. Pulmonary mechanics and energetics of normal non–ventilated low birthweight infants. *Pediatr Pulmonol.* (1990) 8:89–95. 10.1002/ppul.1950080206 2352789

[15] Huckstadt T Foitzik B Wauer RR Schmalisch G. Comparison of two different CPAP systems by tidal breathing parameters. *Intensive Care Med.* (2003) 29:1134–40. 10.1007/s00134-003-1785-0 12774158

