# Supplementary material for: The Effect of a Higher Bias Gas Flow on Imposed T-Piece Resistance and Breathing in Preterm Infants at Birth
Source: Front Pediatr. 2022 Mar 17;10:817010. doi: 10.3389/fped.2022.817010 (PMC8968754; doi:10.3389/fped.2022.817010)
Supplement: Supplementary file 1 [file Data_Sheet_1.PDF]

Supplement 1 Calculations

The resistance of the PEEP-valve can be calculated by using Ohm's law, where resistance is equal to a pressure difference divided by the flow, as explained in the main article. In addition, all calculations are based on the set-up shown in Figure 1.

In case the mask is occluded (Qm=0), the resistance is equal to CPAP divided by the bias flow (Qb).

$$(1) Rv = \frac{\Delta P}{Qv} = \frac{(Pd - Pat)}{Qb + Qm} = \frac{(CPAP - 0)}{Qb + 0} = \frac{CPAP}{Qb}$$

In case the infant is breathing, the resistance is equal to the pressure in the device (which will fluctuate because of the breathing), divided by both flows.

$$(2)\ Rv = \frac{\Delta P}{Qv} = \frac{(Pd - Pat)}{(Qb + Qm)} = \frac{Pd}{(Qb + Qm)}$$

Rewriting the latter formula results in a formula to determine the pressure in the device:

$$(3) Pd = Rv(Qb + Qm) = RvQb + RvQm$$

The inspiratory and expiratory flow through the mask (Qm) can be derived from Ohm's law as well. In this case Qm is equivalent to the pressure difference between the device (Pd) and the alveoli (Pal), divided by the resistance of the airways (Raw).

$$(4) Qm = \frac{\Delta P}{Raw} = \frac{(Pal - Pd)}{Raw}$$

Combining this with formula 3 results in:

$$(5) Qm = \frac{\Delta P}{Raw} = \frac{(Pal - Pd)}{Raw} = \frac{Pal - (RvQb + RvQm)}{Raw} = \frac{Pal - RvQb - RvQm}{Raw}$$

$$(6)\ Qm * Raw = Pal - Rv * Qb - Rv * Qm$$

$$(7)\ Qm * Raw + Rv * Qm = Pal - Rv * Qb$$

$$(8)\ Qm(Raw + Rv) = Pal - Rv * Qb$$

$$(9)\ Qm = \frac{Pal - Rv * Qb}{Raw + Rv}$$

From formula 1 we know that:

$$(10)\ Rv = \frac{CPAP}{Qb}, \qquad so\ Rv * Qb = CPAP$$

Adding that in formula 9 results in a formula showing the factors that influence Qm.

$$(11)\ Qm = \frac{Pal - CPAP}{Raw + Rv}$$
